# The Difference in Thigh Circumferences Between 5 and 10 cm Proximal to the Suprapatellar Border Serving as an Indicator of Joint Effusion in Patients With Knee Osteoarthritis: A Cross-Sectional Study

**DOI:** 10.7759/cureus.89123

**Published:** 2025-07-31

**Authors:** Kunihiro Onishi, Shigeharu Tanaka, Atsutoshi Maki, Shinichi Taniguchi, Hiroyoshi Iwaki, Yasushi Miura

**Affiliations:** 1 Department of Rehabilitation, Osaka Orthopedic Hospital, Osaka, JPN; 2 Department of Rehabilitation, Faculty of Health Science, Tokyo Kasei University, Sayama, JPN; 3 Department of Orthopedic Surgery, Osaka Orthopedic Hospital, Osaka, JPN; 4 Department of Rehabilitation Science, Kobe University Graduate School of Health Sciences, Kobe, JPN

**Keywords:** joint effusion, knee osteoarthritis/ koa, knee swelling, suprapatellar bursa, ultrasonography (usg)

## Abstract

Objective

Knee effusion is a clinically significant symptom associated with pain and functional impairment in patients with knee osteoarthritis (KOA). This study aimed to investigate whether the difference in thigh circumferences measured at two distinct proximal sites could serve as a useful indicator of “knee swelling,” particularly “joint effusion,” in KOA patients. In this study, knee swelling was assessed using thigh circumference, and joint effusion was quantitatively measured using ultrasonography.

Methods

A total of 176 KOA patients who were admitted for total knee arthroplasty between June 2023 and June 2024 were included. Preoperative assessments involved measuring the thigh circumference 5 and 10 cm proximally from the superior border of the patella using a tape measure, and quantifying suprapatellar pouch area as an indicator of joint effusion using sagittal ultrasonographic imaging. The association between the suprapatellar bursa area and femoral circumference at 5 and 10 cm proximal to the suprapatellar border, and the difference between these circumferences was determined using Spearman’s correlation. Additionally, A simple linear regression analysis was performed, with thigh swelling as the independent variable and joint effusion as the dependent variable. Statistical significance was set at p < 0.05.

Results

Data from 96 patients (mean age 73.8 ± 8.0 years; Kellgren-Lawrence grade II: 7, III: 50, IV: 39) were analyzed. The mean thigh circumference at 5 and 10 cm proximal to the patella was 40.2 ± 4.4 and 43.8 ± 4.8 cm, respectively, with a mean difference of 3.6 ± 1.2 cm. The average sagittal area of the suprapatellar bursa was 132.4 ± 98.8 mm². The correlation between suprapatellar bursa area and thigh circumference at 5 cm proximal to the superior border of the patella was not significant (r = 0.028, p = 0.789). Similarly, there was no significant correlation between the suprapatellar bursa area and thigh circumference at 10 cm proximal to the superior border of the patella (r = -0.047, p = 0.648). However, a significant correlation was found between suprapatellar bursa area and the difference in thigh circumference measurements at 5 and 10 cm proximal to the superior border of the patella (r = -0.248, p = 0.015). The regression analysis revealed a significant association only between the area of the suprapatellar bursa and the difference in thigh circumference between the two sites (p < 0.05, r = 0.258).

Discussion

While individual thigh circumference measurements showed no significant relationship with joint effusion, the difference between the two measurement sites was significantly associated with effusion. These findings suggest that calculating the difference between thigh circumferences may reduce the influence of muscle mass, fat, and edema, thereby enhancing the estimation of knee effusion.

Conclusion

Assessment of knee swelling using the difference in thigh circumference 5 and 10 cm proximal to the suprapatellar border is a useful and simple method of assessing joint effusion in patients with KOA.

## Introduction

Knee osteoarthritis (KOA) is a progressive joint disease characterized by cartilage degeneration, joint space narrowing, osteophyte formation, and inflammation, all of which significantly contribute to functional limitations and reduced quality of life [1,2]. One of the key clinical manifestations of KOA is joint effusion, which is reflected in synovial inflammation and is associated with worsening symptoms [3]. A previous study indicated that joint effusion directly affects knee pain and range of motion, resulting in impaired activities of daily living [4]. Therefore, it is essential to accurately assess the status of joint effusion in clinical practice.

The suprapatellar bursa plays a crucial role in knee biomechanics and joint stability, particularly in the presence of effusion. Edama et al. [5] reported that the suprapatellar bursa is located in the deep proximal layer of the patella and retains its anatomical position even when joint effusion is present. They also found that the membrane structure of the suprapatellar bursa changes from a unilamellar to a bilamellar structure during knee motion. This mechanism contributes to the dynamic stability of the knee joint and suggests that the morphological changes in the suprapatellar bursa may be closely related to the dynamics of the joint fluid. The suprapatellar bursa, as an extensional structure of the joint capsule, influences the biomechanical environment of the knee joint, and a detailed understanding of its morphological and functional changes is important for understanding the factors contributing to reduced joint function.

Ultrasonography and femoral circumference measurement have been used to assess swelling and joint effusion around the knee joints. Nevalainen et al. [6] reported that ultrasonography has a sensitivity of 97% for detecting knee joint effusion and synovitis in patients with end-stage KOA. Draghi et al. [7] reported that the US correctly identified 78 of 96 patients with joint effusion compared with MRI, with a sensitivity of 81.3% and a specificity of 100%. Furthermore, previous studies [3,8] have shown a strong association between the quantitative assessment of knee joint effusion using ultrasound, pain, and functional impairment. However, despite its usefulness, ultrasonography is limited in its widespread use in routine clinical practice because it requires specialized techniques and expensive equipment [9]. This challenge is particularly pronounced in community healthcare organizations and small clinics, where it is often difficult to perform ultrasonography. Thigh circumference measurement, a simple and widely used assessment method, is an indicator of joint effusion, muscle atrophy, muscle hypertrophy, and soft tissue swelling. Therefore, although previous studies [10,11] have reported an association between thigh circumference and functional impairment, the direct relationship between joint effusion and functional impairment remains unclear.

This study aimed to investigate the usefulness of thigh circumference measurements as an indicator of knee swelling, particularly joint effusion, in patients with KOA. In this study, "knee swelling" was operationally defined as the thigh circumference measured at two points proximal to the patella, while “joint effusion” was objectively quantified using ultrasonographic assessment of the suprapatellar bursa. This study investigated how the measurement of thigh circumference can provide a comprehensive assessment of swelling around the knee joint, as well as how to independently assess joint effusion only from measured femoral circumference.

## Materials and methods

Ethics statement

This study was approved by the Institutional Review Board of our institution before implementing the methods (Osaka Orthopedic Hospital; approval number: KE-2022-01, approval date: December 22, 2022), and all participants gave written informed consent. Although the ethics approval was obtained on December 22, 2022, the actual start of the study was delayed until June 2023 due to various factors, including the impact of the COVID-19 pandemic and the need for training in ultrasound examination procedures.

Participants and study design

**Figure 1:**
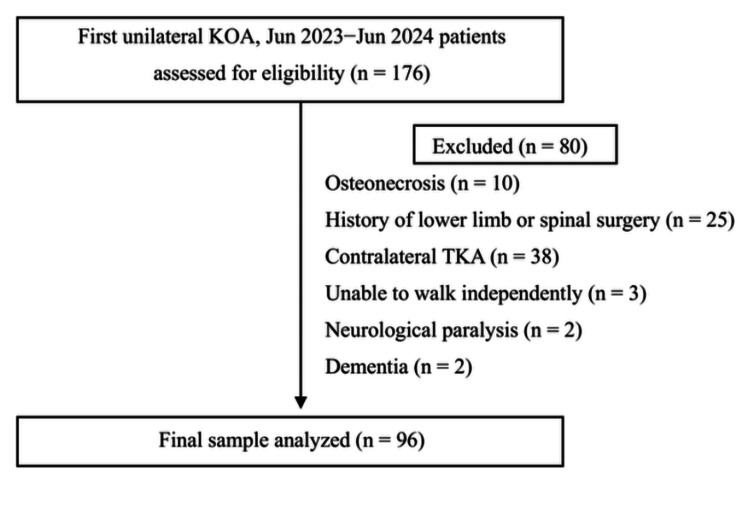
Participant flow diagram KOA, knee osteoarthritis; TKA, total knee arthroplasty

This study was an observational study. Participants were 176 patients with KOA scheduled to undergo primary total knee arthroplasty (TKA) between June 2023 and June 2024. Patients with neurological disorders affecting gait, significant cognitive impairment, psychiatric conditions, or a history of surgery involving the knee or lumbar region were excluded. The detailed flow of patients from enrollment to analysis is shown in Figure 1. Although many participants were initially enrolled during the early phase of recruitment, only 96 participants who met the predefined inclusion and exclusion criteria were included in the final analysis. To minimize the risk of selection bias, all assessments were conducted based on standardized measurement procedures and evaluation criteria throughout the study period. The severity of KOA was assessed using the Kellgren-Lawrence classification, a five-grade system for evaluating KOA severity. A grade of 0 indicates no features of KOA, whereas grade IV represents severe sclerosis and bone deformity, classified as advanced KOA.

Measurement

Knee Swelling - Assessment Using Thigh Circumference

**Figure 2:**
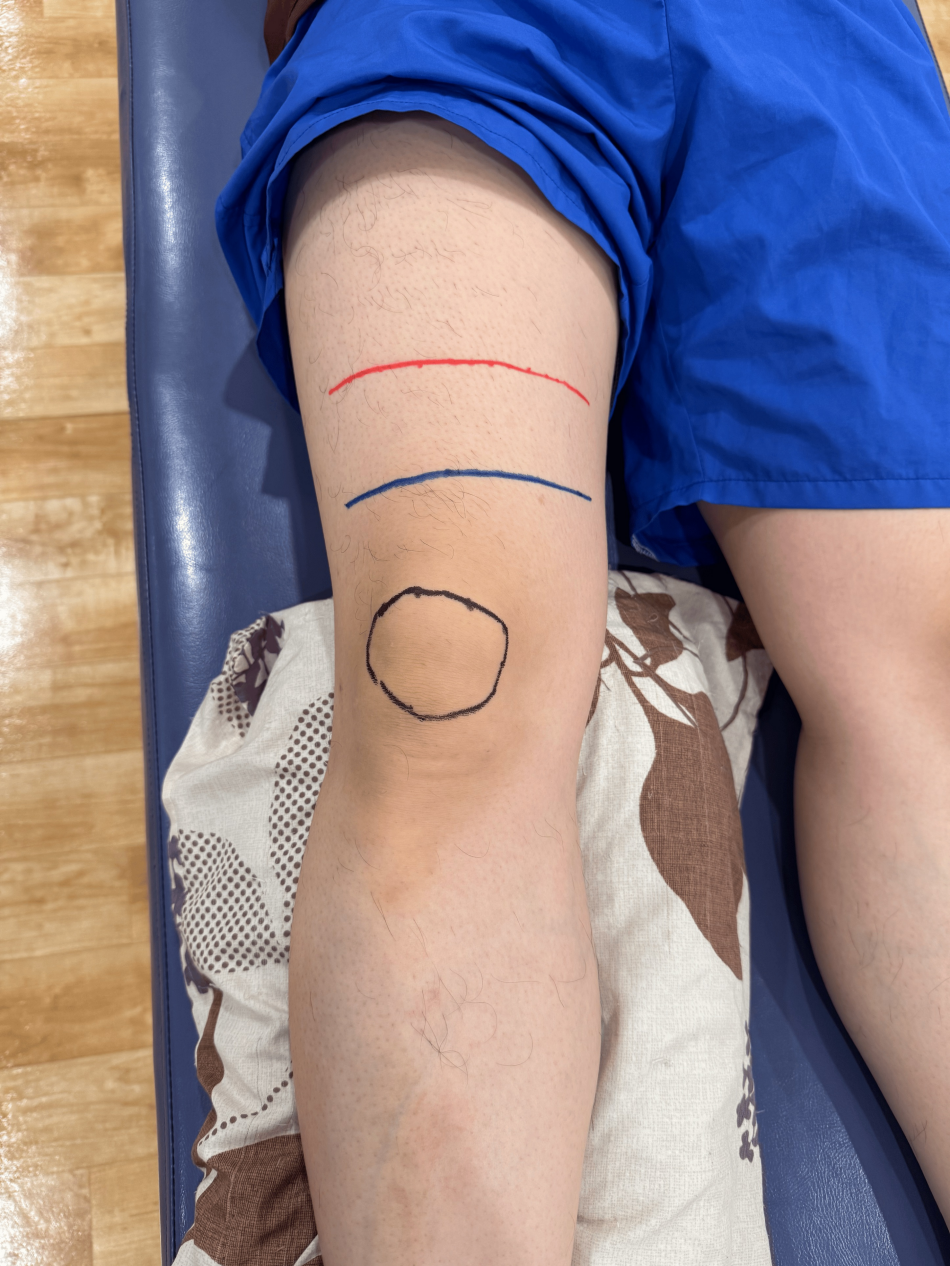
Thigh circumference Knee swelling was evaluated based on the difference in thigh circumference measured at 5 and 10 cm proximal to the superior border of the patella. The blue line indicates 5 cm proximal to the superior border of the patella, and the red line indicates 10 cm proximal to the superior border of the patella.

In this study, the primary outcome was defined as the amount of joint effusion measured by ultrasonography, and knee swelling was evaluated based on the difference in thigh circumference measured at 5 and 10 cm proximal to the superior border of the patella. These two assessments were used to explore their association and the clinical utility of circumference difference as an indicator of effusion. Knee swelling was measured with reference to the study by Kayamori et al. [12]. The reliability of this evaluation method was examined in previous studies [12,13]. Considering previous studies [12-14], body size, and the anatomical location of the suprapatellar bursa, knee circumference measurements were taken at 5 and 10 cm proximal to the superior border of the patella using a tape (Figure 2).

Joint Effusion - Quantitative Evaluation Using Ultrasound

**Figure 3:**
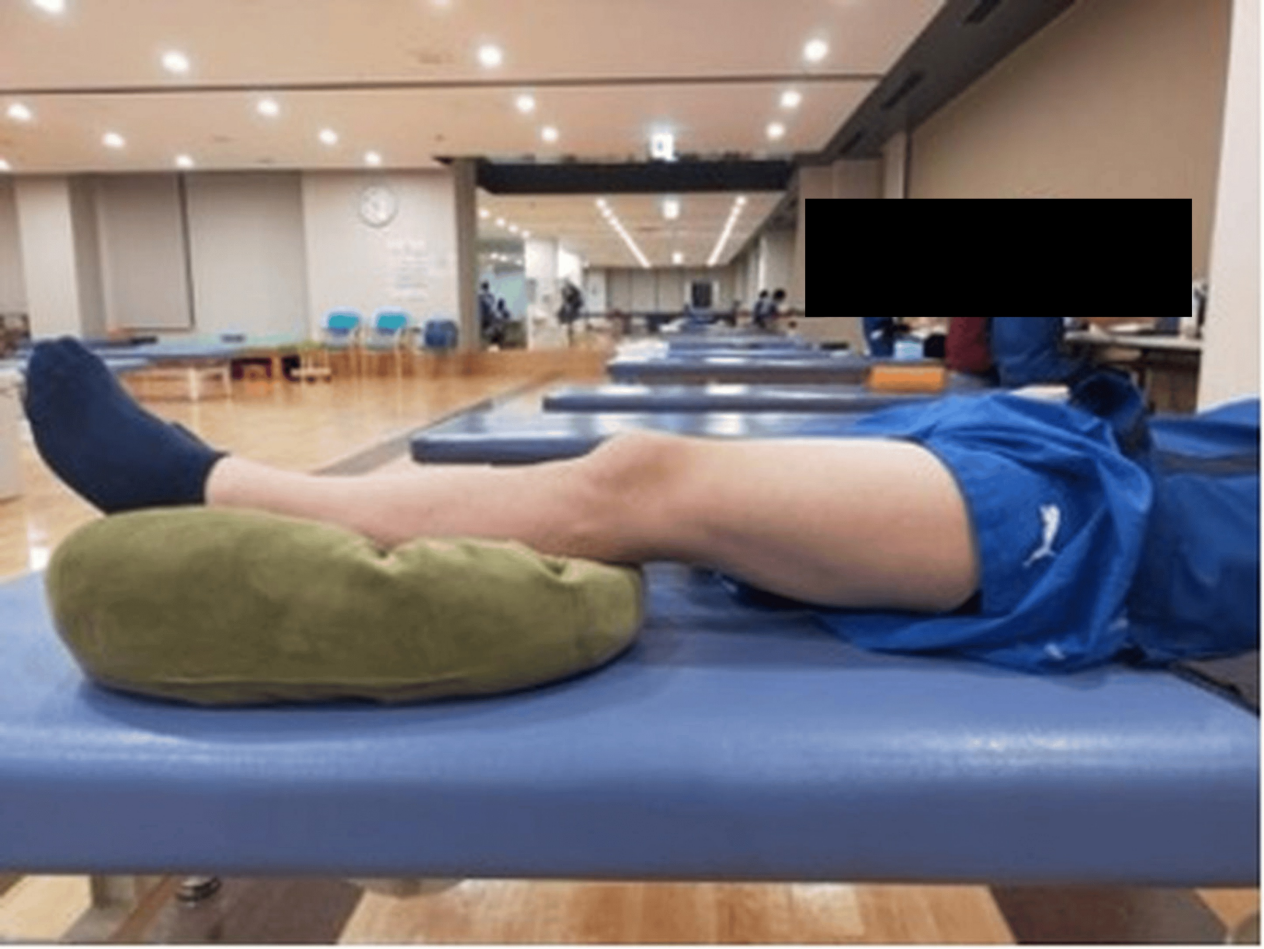
Ultrasound examination positioning Patients were positioned in a relaxed supine position with cushions placed distally to both knees, keeping the knees extended and the lower limbs in a neutral position.

**Figure 4:**
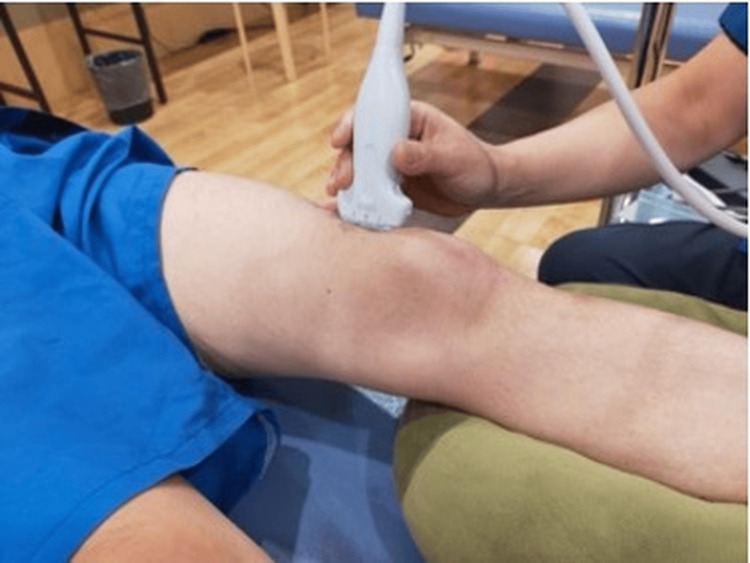
Ultrasound imaging technique of the suprapatellar pouch Ultrasound images of the suprapatellar pouch effusion were obtained by lightly placing a linear probe longitudinally on the suprapatellar pouch.

**Figure 5:**
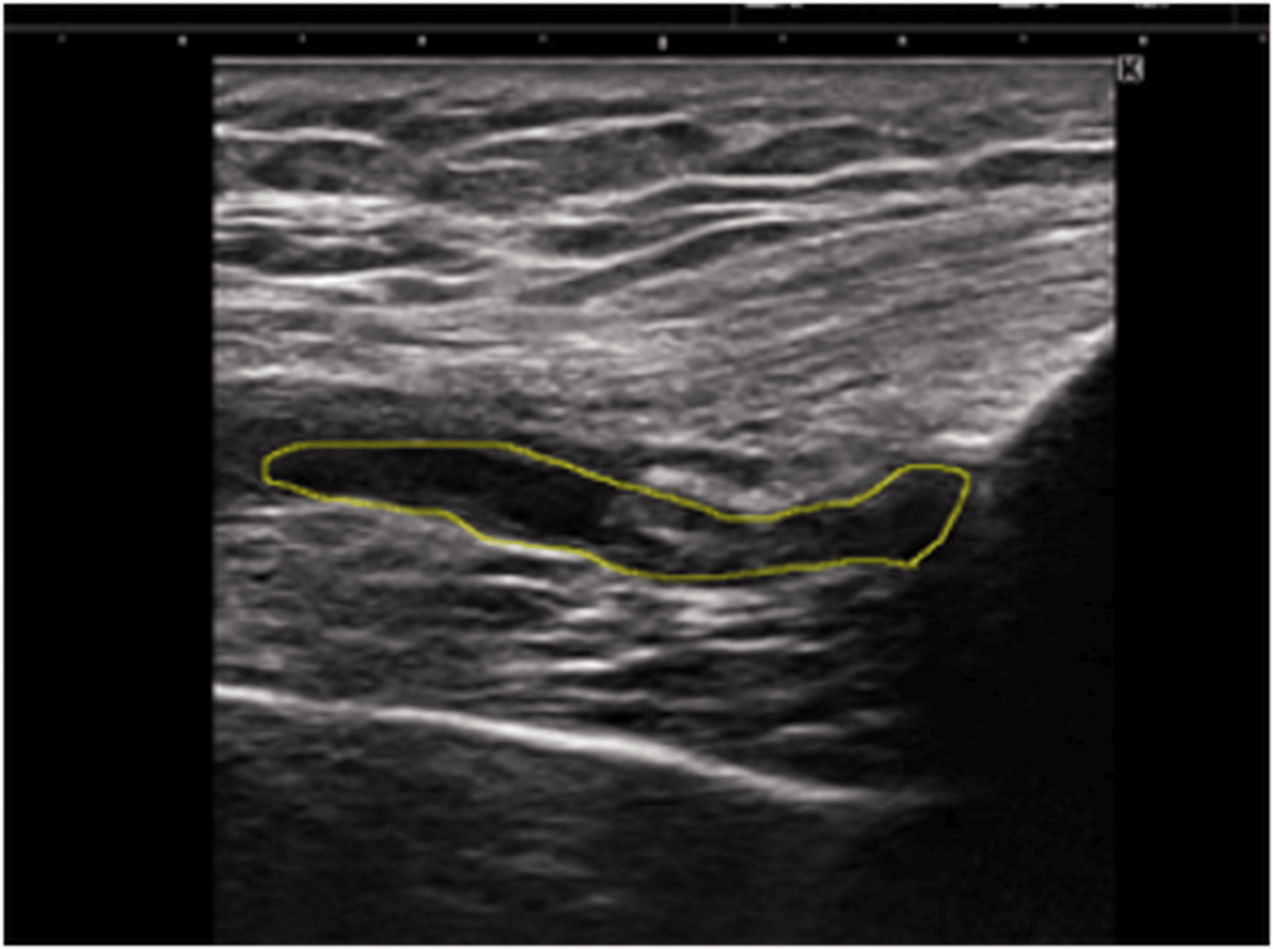
Automatic measurement of suprapatellar effusion area The area of effusion in the suprapatellar pouch (mm²) was automatically calculated by tracing the contour of the effusion, and its cross-sectional area was calculated automatically.

Joint effusion was assessed using ultrasonography (11 MHz; SONIMAGE MX1, Konica Minolta, Inc., Tokyo, Japan), based on previous studies [4,14]. All ultrasound examinations were performed by one examiner: K.O., who had three years of experience in musculoskeletal ultrasound assessment. Ultrasonography was performed to evaluate the amount of effusion in the suprapatellar bursa. The patients were placed in a relaxed supine position with cushions placed distally to both knees, keeping the knees extended and the lower limbs in a neutral position (Figure 3). Ultrasound images of the suprapatellar bursa were obtained by lightly placing a linear probe longitudinally on the suprapatellar bursa (Figure 4). The area of effusion in the suprapatellar bursa (mm²) was automatically calculated by tracing the contour of the effusion, and the cross-sectional area was also calculated automatically (Figure 5). This cross-sectional area served as an indicator of the amount of suprapatellar bursa. Measurements were performed three times, and the average value was used. To confirm the reliability of measuring the area of suprapatellar bursa effusion, the intrarater reliability (intraclass correlation coefficient (ICC) (1,1)) for one examiner testing one subject was 0.991 (95% CI, 0.977-0.997).

Statistical analysis

First, the sample size was calculated using G*Power (Heinrich-Heine-Universität Düsseldorf, Düsseldorf, Germany) with a two-sided α level of 0.05, β of 80%, and a medium effect size (f² = 0.15). The required sample size was estimated as 55 participants. Second, the association between the suprapatellar bursa area and thigh circumference at 5 and 10 cm proximal to the suprapatellar border, and the difference between these circumferences was determined using Spearman’s correlation. Third, statistical analysis was performed using simple regression analysis, with knee swelling as the independent variable and joint effusion as the dependent variable to examine their relationship. The significance was set at less than 5%. Data were analyzed using a statistical software (IBM SPSS Version 22.0 software (IBM Corp., Armonk, NY)).

## Results

Participant characteristics

A total of 96 participants were included in the analysis. The mean age was 73.8 ± 8.0 years, comprising 22 males and 74 females. The average body mass index (BMI) was 24.9 ± 3.8 kg/m². According to the Kellgren-Lawrence classification, seven participants were classified as grade II, 50 as grade III, and 39 as grade IV. The baseline characteristics of the study participants are presented in Table 1.

**Table 1:** Baseline demographic and clinical characteristics of study population (n = 96) Values are means ± standard deviation. TKA, total knee arthroplasty

Variables	Mean ± SD
Age (years)	73.8 ± 8.0
Gender (female/male) (%)	77.0/23.0
Height (cm)	156.0 ± 8.0
Weight (kg)	60.8 ± 11.7
Body mass index (kg/m^2^)	24.9 ± 3.8
Kellgren-Lawrence grade (grade Ⅱ/grade Ⅲ/grade Ⅳ)	7/50/39
Operative side femoro-tibial angle before TKA (degree)	178.8 ± 6.1
Contralateral side femoro-tibial angle before TKA (degree)	177.4 ± 3.5

Knee swelling and joint effusion

Knee swelling was represented by thigh circumference measurements. The mean circumference at 5 cm proximal to the superior border of the patella was 40.2 ± 4.4 cm, while at 10 cm proximal it was 43.8 ± 4.8 cm. The mean difference between the two measurement sites was 3.6 ± 1.2 cm. The mean area of the sagittal section of the suprapatellar bursa was 132.4 ± 98.8 mm² (Table 2).

**Table 2:** Ultrasound measurements of circumferences and joint effusion area in knee osteoarthritis patients Values are means ± standard deviation.

Measurement	Mean ± SD
Circumference (5 cm proximal to patellar upper border)	40.2 ± 4.4 cm
Circumference (10 cm proximal to patellar upper border)	43.8 ± 4.8 cm
Difference in circumference (between 5 and 10 cm proximal)	3.6 ±1.2 cm
Area of joint effusion	132.4 ± 98.8 mm²

The association between knee swelling and joint effusion

The correlation between suprapatellar bursa area and thigh circumference at 5 cm proximal to the superior border of the patella was not significant (r = 0.028, p = 0.789). Similarly, there was no significant correlation between the suprapatellar bursa area and thigh circumference at 10 cm proximal to the superior border of the patella (r = -0.047, p = 0.648). However, a significant correlation was found between suprapatellar bursa area and the difference in thigh circumference measurements at 5 and 10 cm proximal to the superior border of the patella (r = -0.248, p = 0.015) (Table 3).

**Table 3:** Correlation between suprapatellar effusion area and thigh circumference at different thigh levels *Significant association (p < 0.05). The association between the suprapatellar bursa area and thigh circumference at 5 and 10 cm proximal to the suprapatellar border, and the difference between these circumferences was determined using Spearman’s correlation.

Measurement site	Variable comparison	Correlation coefficient (r)	p-value
5 cm proximal to patellar upper border	Suprapatellar effusion area vs. thigh circumference	0.028	0.789
10 cm proximal to patellar upper border	Suprapatellar effusion area vs. thigh circumference	-0.047	0.648
Difference between 5 and 10 cm	Difference in suprapatellar effusion area vs. difference in thigh circumference	-0.248	0.015*

Simple linear regression analysis with knee joint swelling as the independent variable and joint effusion as the dependent variable

A simple linear regression analysis was conducted to examine the relationship between knee swelling, which was treated as an independent variable, and joint effusion. The regression analysis between the thigh circumference measured at 5 cm proximal to the superior border of the patella and joint effusion yielded a β coefficient of 2.213 (p = 0.343). For the circumference measured at 10 cm proximal, the β coefficient was 0.311 (p = 0.884). In contrast, the difference between the 5 and 10 cm proximal circumference measurements showed a significant association with joint effusion (β = -18.440, p = 0.011). These results indicated that only the difference between the two circumference measurements was significantly associated with the area of joint effusion (Table 4).

**Table 4:** Results of simple regression analyses examining the association between thigh circumference and joint effusion area in patients with knee osteoarthritis *Significant association (p < 0.05). Thigh swelling as the independent variable and joint effusion as the dependent variable.

Independent variable	β	p-value	95% CI
Circumference at 5 cm proximal to the superior border of the patella	2.213	0.343	-2.39 to 6.82
Circumference at 10 cm proximal to the superior border of the patella	0.311	0.884	-3.92 to 4.54
Difference between circumferences (5 cm vs. 10 cm proximal)	-18.440	0.011*	-32.60 to -4.28

## Discussion

This study aimed to investigate whether the thigh circumference can serve as a useful indicator for predicting joint effusion in patients with KOA. Specifically, this study focused on the relationship between the thigh circumference measured at 5 and 10 cm proximal to the superior border of the patella and joint effusion, with the aim of evaluating its effectiveness as an objective indicator of swelling in preoperative TKA assessments. The results revealed a significant association between the difference in thigh circumference between the two locations and joint effusion. However, no clear relationship was observed between individual circumference measurements and joint effusion.

Numerous studies have evaluated knee swelling using thigh circumference. For example, Pinsornsak et al. [11] assessed the effects of compression bandaging in patients undergoing TKA by measuring the thigh circumference to evaluate knee swelling. Several studies assessing knee swelling by thigh circumference have been reported in the past. Pinsornsak et al. [11] assessed knee swelling by measuring it using thigh circumference to examine the effect of compression bandaging on TKA patients. Knee swelling was assessed by measuring the perimeter 10 cm above and 10 cm below the superior and inferior borders of the patella, respectively, Kayamori et al. [12] investigated the effectiveness of compression bandages using polyethylene foam pads and evaluated postoperative swelling by measuring the thigh circumference at the superior border of the patella and at 10 and 15 cm proximally. Yu et al. [13] assessed the effects of compression bandaging in post-TKA patients by measuring the circumferences at the superior and inferior borders of the patella and at the midpoint between these two locations. However, none of these studies found a significant relationship between the interventions and knee swelling. Nevertheless, the thigh circumference is widely used in clinical practice to assess lower limb function and activities of daily living.

However, several studies have evaluated joint effusion independently. Chiba et al. [4] demonstrated an association between suprapatellar joint effusion assessed using ultrasonography and knee pain and dysfunction in patients with KOA, suggesting that effusion could negatively affect joint function. In a separate study, Chiba et al. [15] identified joint effusion as a predictor of muscle atrophy progression and found that it was associated with worsening pain and functional decline. Tanaka et al. [14] reported that some individuals perceived knee swelling even in the absence of effusion and tended to experience more severe pain and disabilities. Collectively, these studies [4,14,15] suggest that joint effusion itself may have deleterious effects on lower limb function.

In general, it is difficult to assess thigh circumference independently of the effects of joint effusion. Anat et al. [16] reported that intermuscular fatty tissue is susceptible to changes in body weight and that weight loss causes a marked reduction in thigh muscle area. This suggests that the thigh circumference may be affected by multiple factors, including muscle mass, adipose tissue, edema, and joint effusion. In the present study, there was also no significant association between thigh circumference at a single measurement point and the amount of joint effusion assessed by ultrasonography, possibly because the effects of effusion were masked by multiple other factors. Anatomically, the suprapatellar bursa is a structure located above the patella and continuous with the joint cavity along the distal anterior surface of the femur [17-19]. It is considered oval in shape [20]. Woodley et al. [19] reported that the average length and width of the suprapatellar bursa are 4.6 ± 0.7 and 3.0 ± 0.3 cm, respectively. Thus, the superior margin of the suprapatellar bursa was assumed to be more than 5 cm proximal to the suprapatellar bursa. However, based on previous studies [19], it is likely that it did not reach 10 cm proximal to the suprapatellar bursa. Therefore, it is assumed that the presence of joint effusion increases the circumferential diameter at a site 5 cm proximal to the suprapatellar bursa, whereas the proximal 10 cm site is hardly affected by the effusion itself. It is therefore inferred that by taking the difference in circumference between these two points, other factors such as muscle mass and adipose tissue can be offset, and a clear link with the amount of joint effusion can be captured.

Ultrasonography is an effective imaging modality for visualizing joint effusions and is recognized for its high sensitivity and specificity [21,22]. Repeated use is safe due to the absence of radiation exposure. However, its application requires specialized equipment and technical expertise, limiting its widespread use in clinical settings [23,24]. In contrast, thigh circumference measurement is a simple, reproducible, useful, and practical alternative to routine care. Nonetheless, previous studies such as those by Pinsornsak et al. [11], Kayamori et al. [12], and Yu et al. [13] have employed different anatomical landmarks for circumference measurements, leading to inconsistencies that complicate comparisons across studies. Standardized measurement protocols are essential to accurately assess the impact of joint effusion. To assess the effects of joint effusion, it is important to perform measurements at two sites: one at the suprapatellar bursa and the other proximal to it, as in the present study, 5 and 10 cm above the suprapatellar border. Although this study included only patients with KOA, this measurement approach may also be applicable to other conditions associated with joint effusion, such as rheumatoid arthritis. In this study, the mean area of suprapatellar joint effusion was 132.4 ± 98.8 mm², and the mean difference in thigh circumference between the two measurement points was 3.6 ± 1.2 cm. A previous study by Chiba et al. reported a mean effusion area of 179.3 ± 112.3 mm² in patients with radiographic KOA, which appears slightly higher than that observed in our cohort [4]. This discrepancy may be attributed to differences in disease severity or slight variations in measurement techniques. While reference values for thigh circumference differences are limited in the literature, the measurement protocol used in our study is consistent with previous postoperative swelling evaluations, supporting the appropriateness of our approach.

This study has some limitations. First, the participants were limited to those with KOA scheduled for TKA; therefore, caution should be exercised when generalizing the findings to individuals with early-stage or nonsurgical KOA. Second, joint effusion was measured using ultrasonography at a single longitudinal site aligned with the superior border of the patella, which may not have fully captured the entire effusion within the joint capsule. Third, although the relationship between knee swelling and joint effusion was assessed using a simple regression analysis, a more comprehensive understanding could be achieved by conducting a multivariate analysis that accounted for confounding factors such as age, sex, and BMI.

## Conclusions

These findings suggest that the difference in thigh circumference measured at 5 and 10 cm proximal to the superior border of the patella may serve as a simple and noninvasive indicator for assessing joint effusion in patients with KOA.
